# A case report of X-linked ichthyosis associated with epilepsy due to an *Xp22.31* deletion fragment

**DOI:** 10.3389/fmed.2026.1699866

**Published:** 2026-03-17

**Authors:** Yangfan Qi, Shuangzhu Lin, Yanqiu Zhou, Kai Jiang

**Affiliations:** 1 Changchun University of Chinese Medicine, Changchun, China; 2 Diagnosis and Treatment Center for Children, The Affiliated Hospital of Changchun University of Chinese Medicine, Changchun, China

**Keywords:** ADHD, children, epilepsy, steroid sulfatase, X-linked ichthyosis

## Abstract

**Background:**

X-linked ichthyosis (XLI) is a genetic skin disorder caused by defects in the steroid sulfatase (*STS*) gene, characterized by dry skin and excessive scaling. The majority of patients (90%) have deletions of the *STS* gene.

**Case summary:**

Herein, we report a 5-year-old boy who presented with significant seizures and ichthyosiform skin lesions, along with short stature and attention deficit hyperactivity disorder (ADHD). His skin exhibited an ichthyosiform appearance, diffusely distributed over the entire body. The seizures were characterized by upward gaze deviation and flaccidity of all four limbs, lasting approximately 2 min. Whole-exome sequencing (WES) identified a pathogenic deletion of approximately 1.47 Mb at the Xp22.31 locus in the proband. The father carried the wild-type allele, while the mother was found to have a heterozygous deletion of approximately 1.14 Mb at Xp22.31. This variant was classified as pathogenic according to the American College of Medical Genetics and Genomics (ACMG) guidelines.

**Conclusion:**

We report a rare case of Xp22.31 deletion-associated seizures accompanied by ichthyosis, with concomitant short stature and ADHD. This case further highlights our understanding of the complexity of XLI.

## 1 Introduction

X-linked ichthyosis (XLI) is a genetic disorder of skin keratinization caused by defects in the steroid sulfatase (*STS*) gene, characterized by dark-brown, scaly skin. Beyond dermatological manifestations, XLI has been linked to various neurological conditions, including epilepsy, ADHD, and autism spectrum disorder (ASD) (1, 2). These associations suggest that XLI is not merely a skin condition but may involve broader neurobiological processes in its pathogenesis.

Specifically, the *STS* gene encodes the steroid sulfatase enzyme, a membrane-bound microsomal protein that hydrolyzes various steroid sulfates. In the skin, STS converts cholesterol sulfate to cholesterol; its deficiency leads to the accumulation of cholesterol sulfate in the stratum corneum, inhibiting normal desquamation and resulting in the characteristic scaly skin. Beyond its role in keratinization, STS is expressed in the brain, where it regulates the metabolism of neurosteroids such as dehydroepiandrosterone sulfate (DHEAS). Elevated DHEAS levels resulting from STS deficiency can act as a GABA-A receptor antagonist, thereby increasing neuronal excitability and potentially contributing to neurodevelopmental comorbidities, including epilepsy and ADHD (3, 4).

Research indicates that patients with X-linked ichthyosis exhibit a significantly higher incidence of epilepsy than the general population, which may be associated with microdeletions in the Xp22.31 region. This chromosomal segment contains not only the *STS* gene but also other neurodevelopmental genes, such as the variably charged X chromosome (*VCX*) family (including *VCX*, *VCX2*, and *VCX3B*), pseudouridine 5′-phosphatase (*PUDP*), and patatin-like phospholipase domain-containing 4 (*PNPLA4*) (1, 5, 6). Notably, studies have shown that individuals with Xp22.31 deletions exhibit elevated epilepsy rates, often accompanied by mild cutaneous manifestations (5). Furthermore, another study revealed that XLI patients demonstrated a 13% prevalence of epilepsy and 30% prevalence of ADHD, further supporting the established link between XLI and neurological disorders (2).

Previous clinical reports indicate that epilepsy in individuals with Xp22.31 deletions typically manifests during early childhood, with seizure types ranging from generalized tonic–clonic to focal seizures (1, 5). Notably, febrile seizures are frequently documented as the initial neurologic presentation, similar to the clinical course observed in our patient (6). Regarding management, most patients are treated with conventional anti-epileptic drugs (AEDs), such as valproic acid, levetiracetam, or carbamazepine (5, 6). Treatment outcomes are generally favorable, with a high success rate in achieving seizure control through monotherapy, although long-term follow-up is essential due to the risk of subclinical discharges or neurodevelopmental comorbidities (1, 5).

In specific case studies, some patients with X-linked ichthyosis (XLI) exhibit epilepsy, intellectual disability, and other neuropsychiatric symptoms that may be associated with deletion or mutation of the *STS* gene (7, 8). For instance, one study reported two brothers with XLI caused by a frameshift mutation in the *STS* gene, accompanied by epilepsy, short stature, and reduced bone density (7). Additionally, another case report indicated that XLI patients may present with cerebellar ataxia and neuropsychiatric symptoms, suggesting that XLI could be linked to broader neurological dysfunction (8).

## 2 Case presentation

Chief complaint: A 5-year-old boy presented to our hospital following a recent convulsion. He also exhibited behavioral symptoms and ichthyosis.

The patient had experienced two febrile seizures at 3 years of age, characterized by limb shaking, upward and rightward gaze deviation, opisthotonos, clenched fists, and generalized limb stiffness, each resolving spontaneously within 3–5 min. No systematic evaluation or treatment had been pursued by the parents. One day prior to admission, shortly after taking a hot bath, the child felt drowsy and uncomfortable, followed by upward gaze deviation, diminished pupillary light reflex, and flaccid weakness in all four limbs. The episode lasted approximately 2 min and resolved spontaneously; body temperature was normal.

Current status: The child was seizure-free on admission. He exhibited symptoms of inattention, hyperactivity, impulsivity, and learning difficulties. Formal neurodevelopmental assessments were conducted to evaluate his status. Cognitive testing using the Wechsler Preschool and Primary Scale of Intelligence (WPPSI-IV) yielded a Full Scale Intelligence Quotient (FSIQ) of 84, placing the patient in the low-average range. Additionally, the SNAP-IV scale confirmed significant symptoms of inattention and hyperactivity (detailed in Table 1).

**Table 1:** Results of formal neurodevelopmental assessments.

Assessment tool	Domain/Subscale	Score/Result	Clinical interpretation
WPPSI-IV	Full scale IQ (FSIQ)	84	Low average
SNAP-IV	Inattention	2.40 (Avg)	Severe abnormality
	Hyperactivity/impulsivity	1.70 (Avg)	Mild abnormality
	Oppositional defiant	14 (Total)	Mild abnormality

Physical Examination: On admission, the patient’s height was 100 cm (−2SD) and his weight was 20 kg (+1SD). He was conscious, alert, and responsive with age-appropriate speech. Dermatological examination revealed generalized dry, rough skin with a dark, ichthyosiform appearance, particularly prominent on the abdomen and neck. Polygonal scales with desquamation were noted, and multiple patchy, deep-brown hyperpigmented macules were present on the skin surface. No corneal opacities were observed on fundoscopic examination. No cardiac murmurs or arrhythmias were noted during auscultation. Bilateral testes were descended into the scrotum. Cardiopulmonary, abdominal, and precordial examinations showed no significant abnormalities. Neurological examination indicated normal muscle strength and tone in all limbs. Bilateral knee jerks and Achilles tendon reflexes were symmetrical and present, with negative Babinski, Brudzinski, and Kernig signs.

Birth and Developmental History: Detailed history revealed he was the first child, born full-term via cesarean section due to oligohydramnios and fetal distress. Birth weight was 3.20 kg, and birth length was 50 cm. No abnormalities were noted at birth. The mother was of advanced maternal age and had a history of gestational diabetes mellitus during pregnancy, reportedly well-controlled with diet and exercise. She denied any use of special medications or exposure to radiation.

Family history: The patient’s father and mother are healthy; there was no family history of febrile seizures, epilepsy, developmental delay, or other genetic disorders.

Routine clinical examinations: Laboratory tests: Complete blood count, liver function tests, renal function tests, electrolyte panel, and thyroid function tests revealed no significant abnormalities.

Imaging: Echocardiography and abdominal ultrasound revealed no significant abnormalities.

**Figure 1:**
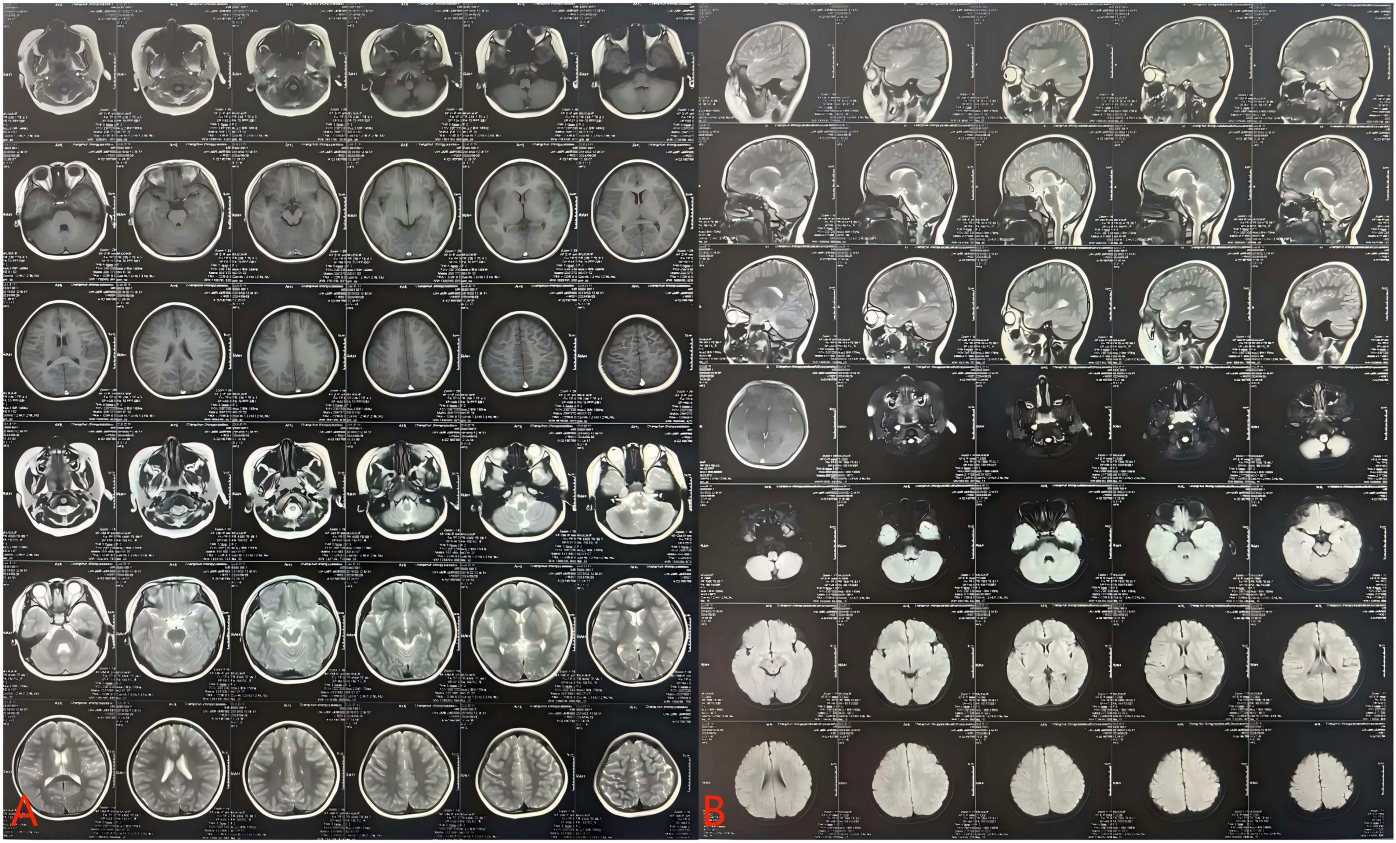
Child’s MRI.

**Figure 2:**
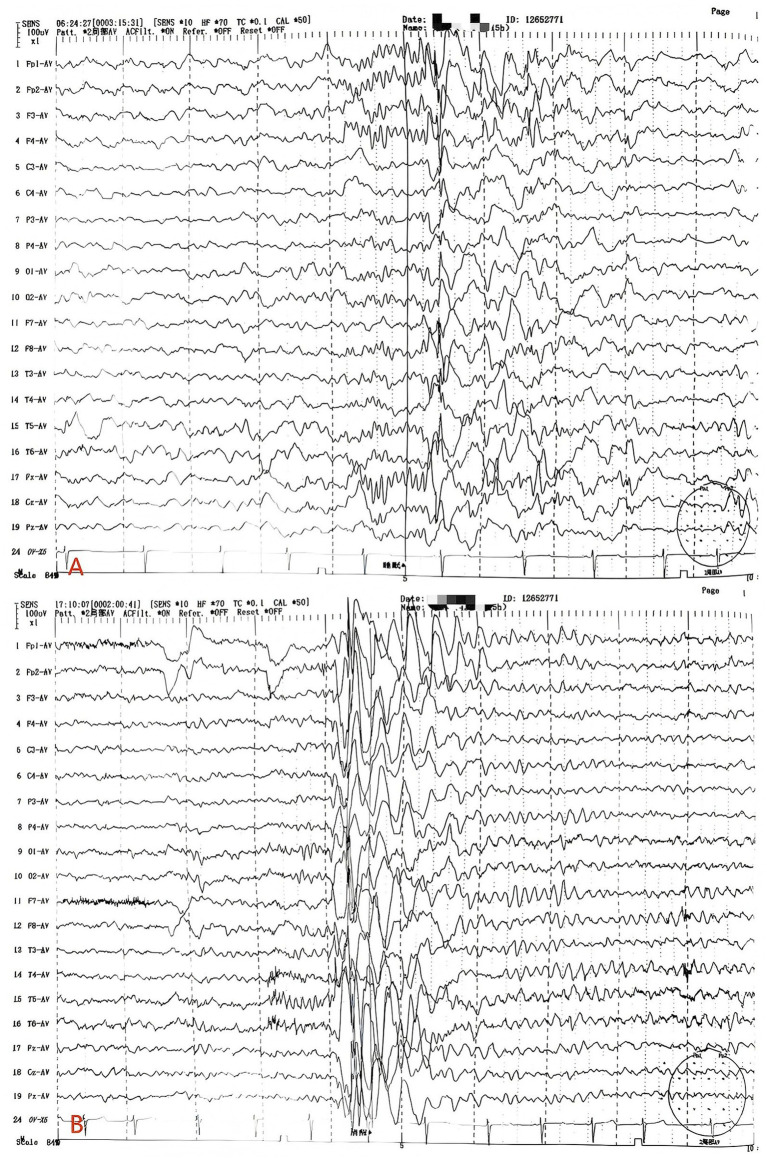
**(A)** Discharge during sleep in children. **(B)** Discharge during the wakefulness of children.

**Figure 3:**
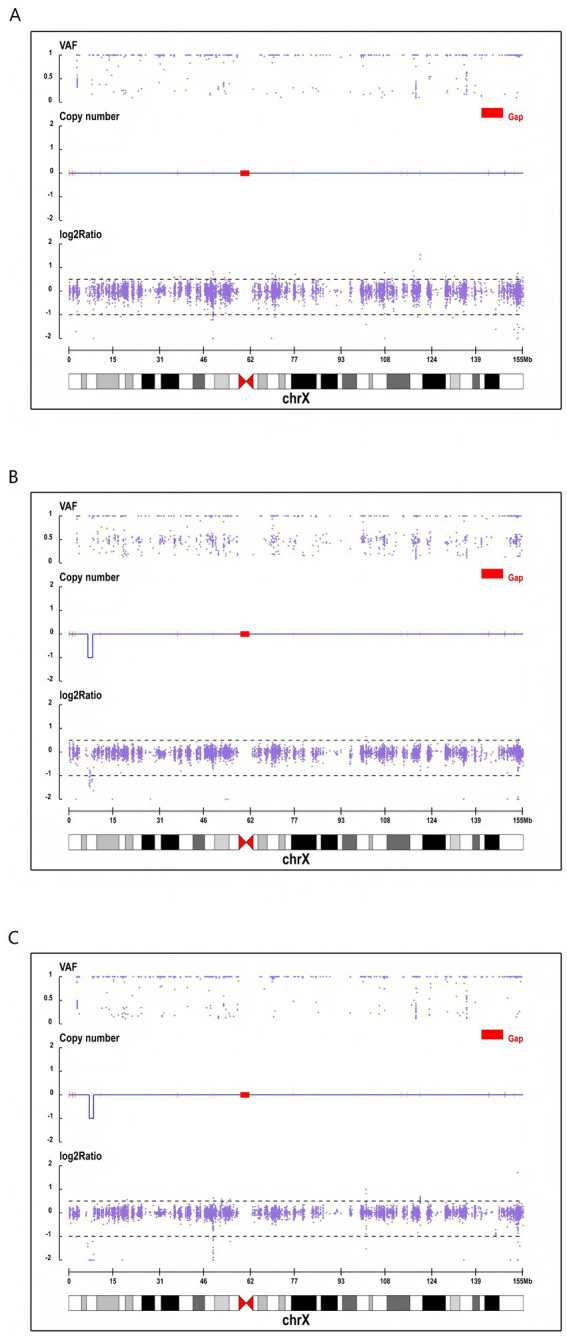
Genetic analysis of the Xp22.31 microdeletion in the family trio. **(A)** CNV plot of the father, showing a normal (wild-type) pattern on the X chromosome. **(B)** CNV plot of the mother, indicating a heterozygous deletion at the Xp22.31 locus, with a characteristic VAF distribution at 0.5. **(C)** CNV plot of the proband, demonstrating a hemizygous deletion at the Xp22.31 region, characterized by a deep copy number dip and the absence of VAF at the 0.5 level.

Brain MRI: No significant abnormalities were detected (Figure 1). Video-EEG: Pediatric EEG monitoring showed abnormal findings characterized by generalized or predominantly bifrontal spikes, spike-and-slow waves, and slow waves intermixed with spikes (detailed in Table 2 and Figures 2, 3).

**Table 2:** Translation of the original video electroencephalogram (VEEG) report.

Section	Content of the original report
Basic information	Patient: Male, 5 years and 11 months old; medication: no anti-seizure medications (ASMs) used; Monitoring: 15-h video-EEG.
Background activity	Awake and quiet state: Bilateral occipital 8–9 Hz, low-to-medium amplitude α rhythm, mixed with a small amount of 6–7 Hz low-to-medium amplitude θ waves. Poor modulation and symmetry. The α rhythm was fully suppressed upon eye-opening.
Hyperventilation	Significant increase in slow waves in all leads.
IPS test	Intermittent photic stimulation (IPS) showed no related abnormal waves during eye-opening, eye-closing, or eye-closed states.
Sleep period	Sleep waves and sleep cycles are generally normal.
Interictal	Frequent medium-to-high amplitude spikes, spike-slow waves, and slow waves, predominantly in the bilateral frontal and prefrontal regions, or presented as generalized discharges.
Ictal period	No clinical seizure events were recorded during the monitoring period.
Impression	Abnormal VEEG: Frequent multifocal and generalized spikes, slow waves, and spike-slow waves, primarily involving the bilateral frontal regions.

Genetic analysis: Family-based whole-exome sequencing (WES) identified a pathogenic hemizygous deletion of approximately 1.47 Mb at the Xp22.31 region in the proband. Based on the GRCh38 (hg38) assembly, the genomic coordinates of this deletion are chrX:7121498–8586755. The deleted region encompasses the STS, PUDP, PNPLA4, VCX, VCX3B, VCX2, MIR4767, and MIR651 genes. Family validation confirmed that the father carried the wild-type allele, while the mother harbored a heterozygous deletion of approximately 1.14 Mb at the same locus (Figure 3). According to the American College of Medical Genetics and Genomics (ACMG) guidelines, this copy number variation (CNV) was classified as pathogenic. Notably, a comprehensive analysis of the WES data did not reveal any other pathogenic or likely pathogenic single-nucleotide variants (SNVs) or small indels that could potentially contribute to the child’s complex phenotype, including epilepsy and ADHD.

Final diagnosis: X-linked ichthyosis.

Therapeutic intervention and outcome: Following admission, based on the clinical seizures and abnormal EEG findings, the patient was initially prescribed oral levetiracetam at a dose of 10 mg/kg/day. While his clinical condition stabilized without further seizures, the persistence of frequent interictal discharges on follow-up EEG prompted a gradual adjustment of the dosage to 20 mg/kg/day (administered in two divided doses) to ensure optimal neurological stability. Concurrently, emollients and moisturizers were prescribed for the skin condition. Although the patient met the diagnostic criteria for ADHD, pharmacological intervention for neurobehavioral symptoms was deferred to prioritize the stabilization of his neurological status and avoid potential drug-to-drug interactions. Instead, a conservative management approach focusing on sensory integration and concentration training was implemented.

During the subsequent 20-month treatment period and a 1-year follow-up after medication discontinuation, the patient remained completely seizure-free. Notably, his height velocity remained stable at 3–4 cm/year throughout the follow-up period, indicating favorable systemic stability following seizure control. Apart from persistent symptoms of inattention and hyperactivity, no other significant neurodevelopmental or motor issues were identified. The patient will continue to be closely monitored for both neurobehavioral and neurological progress.

## 3 Discussion

XLI is a genetic skin disorder caused by defects in the *STS* gene, characterized by dry skin and excessive scaling. Research indicates that XLI not only affects the skin but may also be associated with various neurobehavioral disorders. For example, one study found that patients with XLI exhibited significantly higher rates of ADHD and autism spectrum disorder, which could be linked to *STS* gene deficiency (3). Furthermore, RNA sequencing and lipidomics studies have revealed that genes related to epidermal differentiation and lipid metabolism are markedly downregulated in keratinocytes from XLI patients, potentially contributing to both their skin manifestations and systemic symptoms (4).

Clinically, patients with XLI typically present with generalized dryness and scaling of the skin, particularly on the extensor surfaces of the limbs and the sides of the torso (9). However, XLI is well-documented to be associated with systemic extracutaneous manifestations, including cryptorchidism, corneal opacities, and cardiac arrhythmias (6, 9). In our case, thorough clinical screening—including physical palpation, fundoscopic examination, and cardiac auscultation—yielded negative findings for these conditions. This suggests a predominantly dermatological and neurological phenotype in this patient. Previous studies indicate that XLI patients exhibit higher rates of neuropsychiatric disorders, including epilepsy and ADHD (2).

In terms of diagnosis and treatment, XLI is typically diagnosed based on clinical manifestations and genetic testing. Fluorescence *in situ* hybridization (FISH) analysis is an effective diagnostic tool for detecting common *STS* gene deletions (10). Current treatments for XLI primarily focus on skin care, including the use of moisturizers and keratolytic agents (9). However, new therapeutic approaches are being actively explored, such as a novel topical isotretinoin formulation, TMB-001, which has demonstrated promising safety and efficacy in clinical trials (11).

Furthermore, genetic counseling and prenatal diagnosis of XLI have gained increasing attention. Non-invasive prenatal screening (NIPS) can effectively detect copy number variations on the maternal X chromosome, thereby guiding prenatal diagnosis of XLI (12). In genetic counseling, understanding the gene mutation spectrum and the clinical manifestations of XLI is crucial for developing personalized management strategies (6).

In conclusion, XLI is not only a skin disease but may also be associated with various neurological and behavioral disorders. Through in-depth research on its pathogenesis and clinical manifestations, we can provide patients with more comprehensive diagnoses and treatment plans, as well as establish an important foundation for genetic counseling and prenatal diagnosis.

Although our patient was initially admitted for “convulsive seizures,” subsequent evaluation revealed classic ichthyosiform skin lesions, inattention, and learning difficulties. Further investigations demonstrated abnormal epileptiform discharges on EEG. The multi-system involvement raised suspicion of an underlying genetic mutation.

Whole-exome sequencing (WES) identified a pathogenic deletion of approximately 1.47 Mb at *Xp22.31*, classified as “Pathogenic” according to the ACMG guidelines. The deleted region encompassed the *STS*, *HDHD1 (PUDP)*, *PNPLA4*, *VCX*, *VCX3B*, *VCX2*, *MIR4767*, and *MIR651* genes. Based on the clinical phenotype, the patient was definitively diagnosed with XLI.

Considering the patient’s complex clinical phenotype, a customized multidisciplinary management plan was implemented. First, levetiracetam was selected as the first-line antiepileptic therapy, initiated at 10 mg/kg/day and titrated to a maintenance dose of 20 mg/kg/day. This titration was guided not only by achieving a seizure-free clinical state but also by the persistence of interictal discharges on follow-up EEG, underscoring the vital role of continuous electrophysiological monitoring in ensuring neurological stability. Regarding comorbid ADHD, although formal assessments using the SNAP-IV and WPPSI-IV (FSIQ 84) confirmed the diagnosis, we opted for a strategic conservative approach. Pharmacotherapy was deferred to prioritize seizure stabilization and minimize potential drug-to-drug interactions. Instead, non-pharmacological interventions, including sensory integration and concentration training, were provided, establishing a stable baseline for the patient’s neurodevelopmental progress.

Regarding the short stature (height −2 SD) observed in our patient, while isolated STS deficiency is traditionally characterized by cutaneous symptoms, growing evidence suggests that growth impairment is an integral part of the clinical spectrum of Xp22.31 microdeletion syndrome (6, 7). This region functions as a contiguous gene syndrome locus. Although the SHOX gene (located at Xp22.33) was not involved in this 1.47 Mb deletion, the loss of adjacent genes or regulatory elements is likely to contribute to developmental and growth delays. Therefore, the patient’s short stature should be recognized as a phenotypic expansion of the Xp22.31 microdeletion, necessitating long-term auxological monitoring in addition to neurological care.

Furthermore, the significance of long-term, multidimensional follow-up in Xp22.31 deletion cases cannot be overemphasized. Our 20-month treatment and 1-year post-medication monitoring demonstrated that the patient remained completely seizure-free and that his ichthyosiform skin lesions were successfully controlled with consistent topical emollient use. Crucially, his growth velocity remained stable at 3–4 cm/year throughout the follow-up period. This auxological stability serves as a key indicator that antiepileptic management did not adversely impact the growth axis—a critical observation in XLI cases, where short stature is a known risk factor. Integrating phenotypic monitoring with persistent follow-up ensures that proactive interventions can be adjusted to optimize both neurological and developmental outcomes.

In summary, we report a case of XLI with comorbid epilepsy caused by an Xp22.31 deletion. The patient presented with ichthyosiform scaling, polygonal desquamation, seizures, inattention, and short stature, but no severe neurodevelopmental deficits. This case enriches the phenotypic spectrum of the disorder. Given that the majority of patients with XLI harbor larger deletions at Xp22.31 spanning multiple genes beyond *STS*, contributing to heterogeneous and complex clinical manifestations, timely evaluation and differential diagnosis are crucial. Integrating phenotypic assessment with genetic testing enables early diagnosis and proactive intervention, thereby optimizing outcomes.

## Data Availability

The original contributions presented in the study are included in the article/supplementary material, further inquiries can be directed to the corresponding author.
